# Editorial: Dissociations between neural activity and conscious state: a key to understanding consciousness

**DOI:** 10.3389/fnhum.2023.1256168

**Published:** 2023-08-02

**Authors:** Joel Frohlich, Julia S. Crone, Pedro A. M. Mediano, Daniel Toker, Daniel Bor

**Affiliations:** ^1^Institute for Neuromodulation and Neurotechnology, University Hospital and University of Tuebingen, Tuebingen, Germany; ^2^Institute for Advanced Consciousness Studies, Santa Monica, CA, United States; ^3^Vienna Cognitive Science Hub, University of Vienna, Vienna, Austria; ^4^Department of Computing, Imperial College London, London, United Kingdom; ^5^Department of Psychology, University of Cambridge, Cambridge, United Kingdom; ^6^Department of Psychology, University of California, Los Angeles, Los Angeles, CA, United States; ^7^Department of Psychology, Queen Mary University of London, London, United Kingdom

**Keywords:** EEG, consciousness, sleep, general anesthesia, disorders of consciousness, biomarkers, coma, unresponsive wakefulness syndrome

## Introduction

In disorders of consciousness (DoC), the behavioral misdiagnosis rate may be as high as 40% (Schnakers et al., 2009). We thus urgently need a way to detect consciousness “from the outside” using objective, physiological biomarkers. Yet abnormal brain activity in severely brain-injured patients doesn't necessarily indicate unconsciousness (Kaplan, 2004; Verellen and Cavazos, 2010). Given this challenge, our editorial explores various strategies for finding true neural markers of (un)consciousness.

## Standard approaches for deriving markers of consciousness

One approach is to observe healthy individuals during temporary unconsciousness (e.g., deep sleep) and see how neural activity changes. Based on these results, other researchers may later look for similar changes in neural activity in DoC patients to infer either consciousness or its absence. In our Research Topic, Aamodt et al. followed this approach by correlating the complexity of electroencephalography (EEG) signals with sleep stages in healthy volunteers (more on this later).

A similar approach is to observe surgical patients during the loss of consciousness induced by general anesthesia and see how neural activity changes with this causal manipulation. In our Research Topic, Sun et al. investigated whether EEG alpha oscillations dynamics recorded from adult and pediatric surgical patients during the initial and maintenance phases of general anesthesia predict alpha band activity during emergence from anesthesia, as well as extubation time. Although the authors' findings were negative, the general approach of studying EEG recordings from surgical patients during general anesthesia (Zhang et al., 2001; Brown et al., 2010), as well as volunteers given anesthesia in the laboratory (Alkire and Miller, 2005; Ńı Mhuircheartaigh et al., 2013; Purdon et al., 2013), may offer insights that translate not only to detecting covert consciousness in DoC, but also during general anesthesia (so-called “anesthesia awareness”) (Sebel et al., 2004; Huang et al., 2018).

However, the gold standard indications of consciousness are volitional, goal-directed behaviors and verbal report: if patients exhibits these behaviors, then we can hardly doubt that they are conscious. In our Research Topic, for instance, a verbal report study of dream content by Aamodt et al. came to the surprising conclusion that the complexity of spontaneous EEG signals, often associated with one's level of consciousness (Sarasso et al., 2021), does not correspond to dream content in the second stage of non-rapid eye movement sleep (NREM2).

In cases of DoC, patients diagnosed with unresponsive wakefulness syndrome (UWS, sometimes referred to as the “vegetative state”) lack verbal reports by definition, even if some of them are covertly conscious (Monti et al., 2010). Of course, that is not to say that we cannot learn from studying heterogeneous DoC patients or contrasting their neural activity with that of healthy volunteers. A preliminary study in our Research Topic by Wu et al., for example, found that whole-brain theta band functional EEG connectivity correlated with functional disability in patients emerging from a minimally conscious state, and that frontoparietal theta connectivity was greater in the patient group than in healthy controls. This study offers a promising lead for measuring the recovery of consciousness with EEG, but much more work is needed to learn to what extent theta band connectivity actually relates to consciousness.

## Markers of consciousness should generalize across diverse regimes of cortical dynamics

So then, if biomarkers of consciousness are best derived from populations outside of DoC who are capable of volitional behaviors, how can we be sure that such biomarkers will generalize to DoC, where cortical dynamics are often abnormal (Kaplan, 2004; Verellen and Cavazos, 2010)? Indeed, one study in our Research Topic by Maschke et al. found that several common measures of consciousness behaved unexpectedly in three UWS patients treated with the general anesthetic propofol. Perhaps more sophisticated approaches are needed to help us identify biomarkers of consciousness that generalize to abnormal EEGs. One such approach is to recruit subjects who exhibit paradoxical EEG dissociations, i.e., both abnormal cortical dynamics *and* volitional behaviors during wakefulness which indicate consciousness (Frohlich et al., 2022, 2023b). These participants are behaviorally responsive (albeit not necessarily verbal), giving us confidence in the labels we use for supervised machine learning. For instance, analyses of EEGs from children with rare 15q genetic disorders, who show dissociations between neural oscillations and conscious state (Frohlich et al., 2020; Saravanapandian et al., 2021), demonstrate that the complexity of EEG signals is a common denominator of consciousness shared with healthy children (Frohlich et al., 2022).

## A framework for studying neural signatures of consciousness

**Figure 1:**
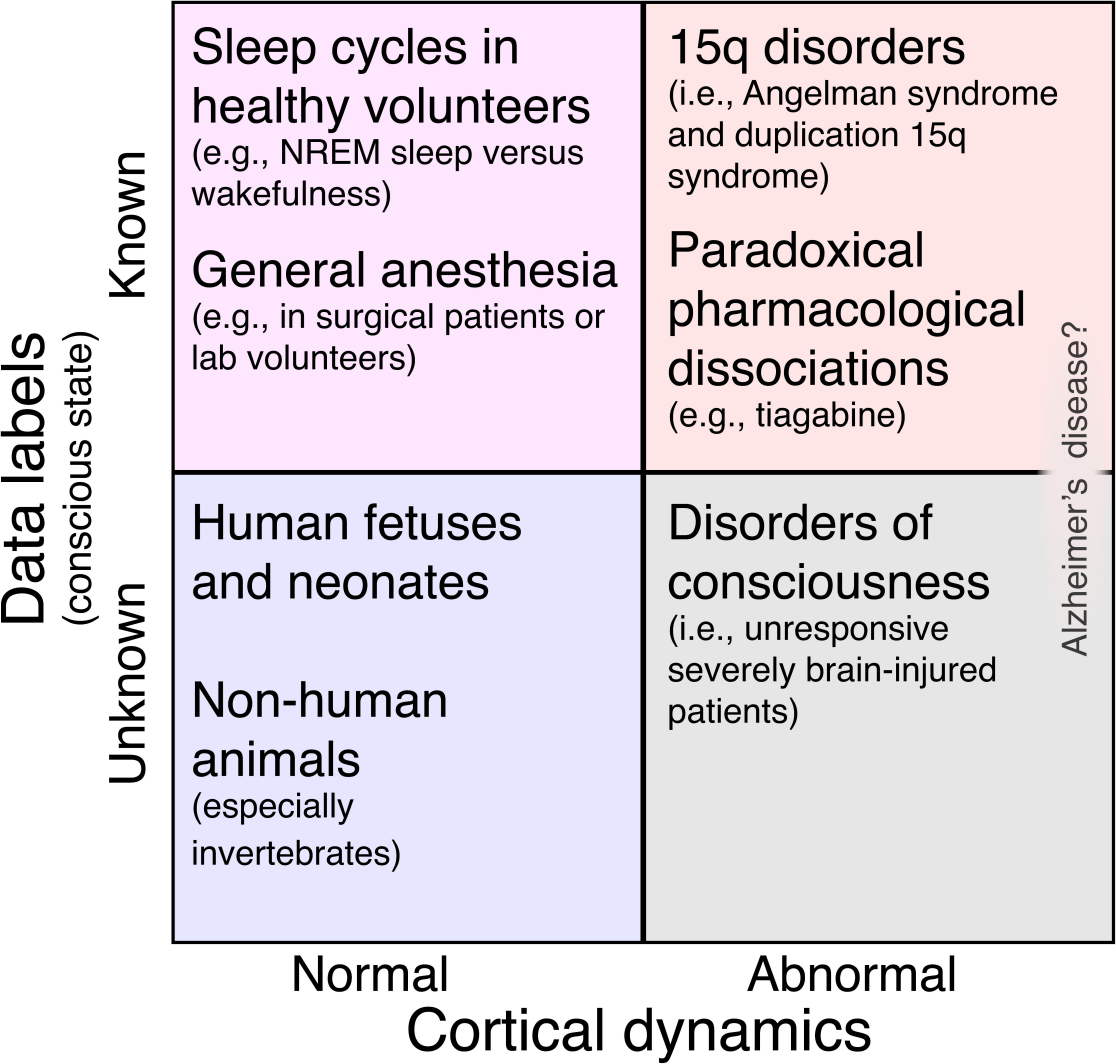
Matrix of study populations for investigating neural biomarkers of consciousness broken down by the presence or absence of abnormal cortical dynamics and the presence or absence of high-confidence data labels to guide supervised machine learning. In the top left quadrant, such labels are available, but the risk of overfitting a binary classifier model to normal EEG features is high. This includes most studies of sleep and general anesthesia [but note that cognitive-motor dissociation sometimes occurs under anesthesia (Sebel et al., 2004); see also (Nilsen et al.)]. In the bottom left quadrant, cortical dynamics are normal (so we risk overfitting), and we don't even know if our subjects possess consciousness. This includes human fetuses and newborns (Frohlich et al., 2023a) and other animal species, especially those that are only distantly related to humans (Birch, 2022). Next, the bottom right quadrant includes subjects with abnormal cortical dynamics whose level of consciousness is uncertain, namely, DoC patients. Finally, the top right quadrant represents our ideal study population: cortical dynamics are abnormal and lack the features that we might otherwise overfit a classifier model to in the top left quadrant. Nonetheless, these participants are behaviorally responsive, giving us labels with which to train a classifier. Besides children with 15q disorders (Frohlich et al., 2022), this quadrant also includes healthy adults challenged with drugs like tiagabine (Nutt et al., 2015; Barnett et al., 2020; Darmani et al., 2021) which result in paradoxical pharmacological dissociations (Frohlich et al., 2023b). Alzheimer's disease (Huntley et al.) can be understood as an ambiguous case that might fall under either the top right or bottom right quadrant.

To summarize, we present a categorization matrix in Figure 1 which describes a framework for studying neural signatures of consciousness. In this framework, most study populations can be placed along two dimensions: cortical dynamics (normal or abnormal) and conscious state labels (known or unknown). While we feel that this matrix is a useful tool for thinking about study designs, not every study will, at present, fit cleanly into it. For example, in our Research Topic, Huntley et al. measured the perturbational complexity index (PCI) (Casali et al., 2013; Comolatti et al., 2019), a highly accurate (Casarotto et al., 2016) surrogate for consciousness, in three patients with severe Alzheimer's disease (AD). Each patient yielded PCI values at or slightly below the threshold for consciousness. Should severe AD be viewed, then, in the upper right quadrant of Figure 1, with PCI values dissociated from consciousness? Or should severe AD be viewed in the lower right quadrant, as a DoC (Huntley et al., 2021) in which PCI accurately tracks consciousness? We hope that once AD and other ambiguous conditions are better understood, the approach in Figure 1 will be broadly applicable.

Finally, we feel humbled by the perspective shared by Nilsen et al. in our Research Topic, who ask, “Are we really ‘unconscious' in unconscious states?” The authors invite us to revisit common assumptions in light of a few key points: behavioral signs and subjective reports of consciousness or its absence are notoriously unreliable and, furthermore, “[s]tates presumed to be unconscious are not always devoid of experience.” Indeed, from birth until death, do the lights of consciousness ever switch off completely?

## Conclusions

We advocate for studying dissociations between neural activity and conscious state as a key to identifying universal markers of consciousness. We look forward to further studies that will challenge not only candidate biomarkers of consciousness, but also our frameworks for understanding them.

## Author contributions

JF: Writing—original draft, Writing—review and editing. JC: Writing—review and editing. PM: Writing—review and editing. DT: Writing—review and editing. DB: Writing—review and editing.
